# Data describing effects of different phosphorus concentrations on growth and chymotrypsin inhibitors in *Microcystis aeruginosa* NIVA Cya 43 using LC-MS

**DOI:** 10.1016/j.dib.2024.110121

**Published:** 2024-02-09

**Authors:** Md. Mohibul Hasan, Zahidul Islam

**Affiliations:** a Department of Environmental Toxicology, Faculty of Biology, Universität Duisburg-Essen, Universitätsstraße 2, 45141 Essen, Germany; b Biozentrum, Institute of Zoology, Faculty of Mathematics and Natural Sciences, Universität zu Köln, Albertus-Magnus-Platz 50923 Köln, Germany; c Bangladesh Fisheries Research Institute, Marine Fisheries and Technology Station, Cox's Bazar - 4700, Bangladesh

**Keywords:** Cyanobacteria, Bioactive secondary metabolites, Protease inhibitors, LC-MS

## Abstract

Cyanobacteria's abundant production of bioactive compounds concerns unselective filter feeders in the aquatic food chain, but the factors driving this production remain poorly understood. Notably, nutrient availability, particularly concerning phosphorus and nitrogen, is believed to be a pivotal determinant of cyanobacterial mass development. In this data investigation, we aimed to explore the influence of dissolved phosphorus (PO_4_^3−^) on the presence of chymotrypsin inhibitors, specifically Cyanopeptolin 954 (CP954) and Nostopeptin 920 (BN920), within *Microcystis aeruginosa* NIVA Cya 43. A carefully controlled 15-day batch culture experiment was conducted, with three distinct phosphate concentrations (30, 50, and 75 µM). Liquid Chromatography-Mass Spectrometry (LC-MS) was employed for quantitative analysis, and the findings underscored the intricate interplay between nutrient availability, particularly phosphorus, and the content of chymotrypsin inhibitors (CP954 and BN920) by Cyanobacteria. More precisely, a significant 53% increase in CP954 content was noticed as the phosphate concentration decreased, revealing the intricate connection between nutrient availability, particularly phosphorus, in Cyanobacteria. Future research should further investigate the impacts of environmental factors, including light intensity and other nutrients like nitrogen, on the content of chymotrypsin inhibitors in Cyanobacteria.

Specifications Table

**Table:** 

Subject	Environmental Toxicology, Aquatic Toxicology, Aquatic chemical ecology
Specific subject area	Cyanobacteria growth, Nutrient variation, Chymotrypsin inhibitors quantification, Info chemical interactions
Data format	Raw and analyzed data
Type of data	Table and Chart
Data collection	Microscopic observation for growth curve; Counting cells using hemocytometer; Quantification of chymotrypsin inhibitors using LC-MS (Ultra high-pressure liquid chromatography system coupled with a couple with an Exactive Orbitrap mass spectrometer).
Data source location	Biozentrum, Institute of Zoology, Faculty of Mathematics and Natural Sciences, Universität zu Köln, Albertus-Magnus-Platz 50923 Köln, Germany and Universität Duisburg-Essen, Universitätsstraße 2 45141 Essen, Germany
Data accessibility	Repository name: Mendeley data Data identification number: 10.17632/f5mtdbkr3r.1 Direct URL to data: http://doi.org/10.17632/f5mtdbkr3r.1

## 1 Value of the Data

• These data will contribute to a deeper understanding of the intricate relationship between nutrient availability, growth dynamics, and protease inhibitor content in cyanobacteria.
• Lower phosphorus concentrations may lead to increased content of chymotrypsin inhibitors, particularly CP954, in *M. aeruginosa* NIVA Cya 43. This finding underscores the role of phosphorus availability in shaping chymotrypsin inhibitor content.
• Utilizing LC-MS for the precise quantitative data of these compounds not only enhances our current understanding but also provides valuable insights to guide future research in this field.

## 2 Background

The data set enables a comprehensive exploration of how nutrient availability, especially phosphorus concentrations, influences the production of chymotrypsin inhibitors in cyanobacteria. This investigation aims to improve our understanding of the complex relationship between nutrient availability and chymotrypsin inhibitor content in cyanobacteria.

**Fig. 1:**
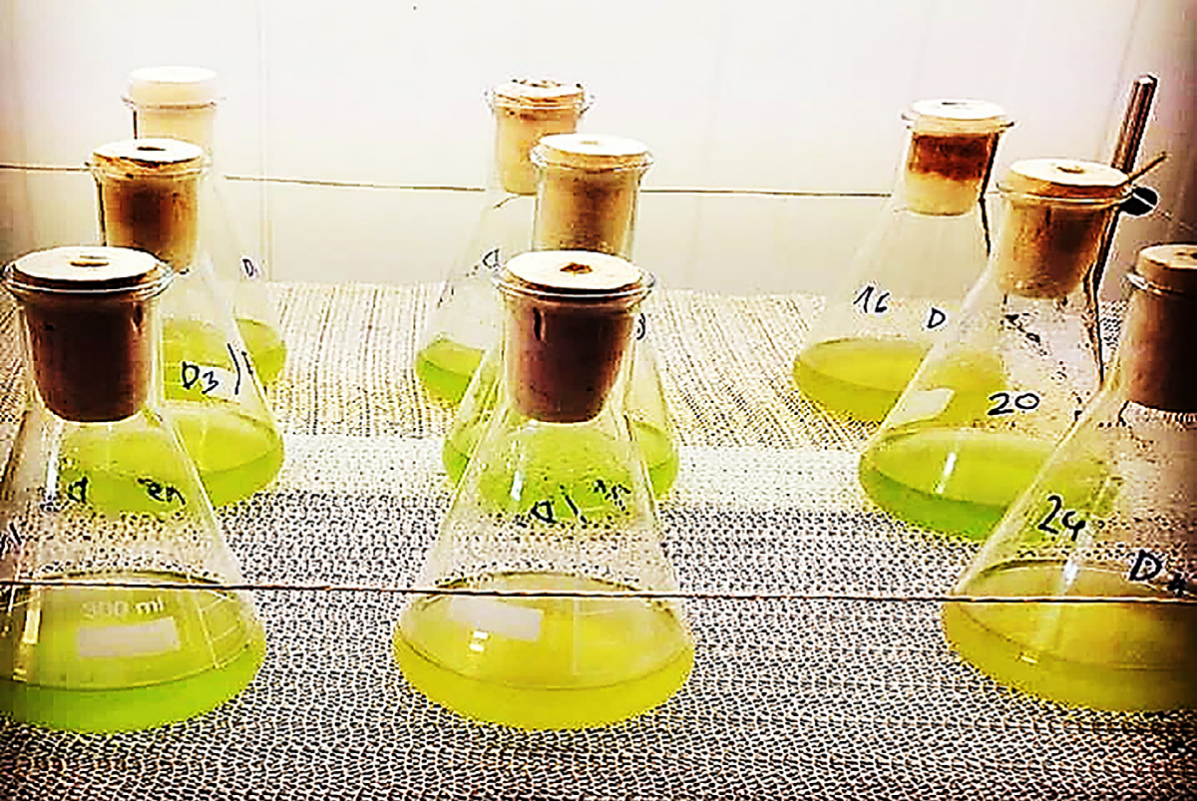
Batch culture of *M. aeruginosa* NIVA Cya 43 in different phosphorus cultures.

The data set serves to facilitate the examination of the relationship between reduced phosphorus concentrations and heightened content of chymotrypsin inhibitors, especially CP954, in *M. aeruginosa* NIVA Cya 43. Through this investigation, it underscores the critical role of phosphorus availability in shaping chymotrypsin inhibitor content and contributes valuable insights to the field of cyanobacterial research. Fig. 1 shows the laboratory setup of the culture unit.

## 3 Data Description

In pre-cultures, the cyanobacterium strain *M. aeruginosa* NIVA Cya 43 was cultivated, and the observed characteristics were summarized in Table 1.

**Table 1:** Characteristics of *Microcystis aeruginosa* NIVA Cya 43 were observed.

Characteristics	Description
Color	Greenish-blue or greenish-brown coloration
Cell shape	Spherical, individual cells
Colony formation	Do not form a colony
Gas Vesicles	Present, regulating buoyancy
Chloroplasts	Absent, containing pigments
Toxin Production	They do not produce microcystins but they produce inhibitors
Size	2 −10 µm in diameter

### 3.1 Growth of *M. aeruginosa* Niva Cya 43 in different phosphorus cultures

**Fig. 2:**
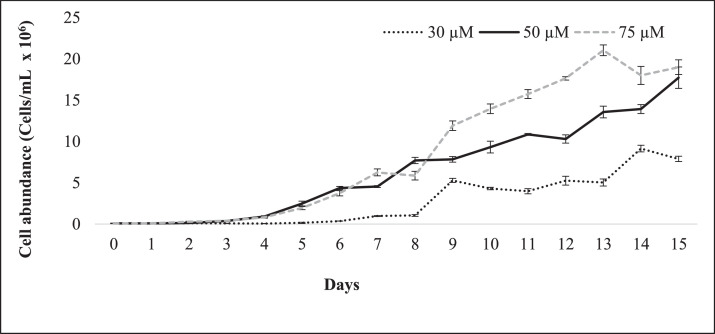
Cell abundance of *M. aeruginosa* NIVA Cya 43 in different time points of the growth experiment in 30 µM,50 µM, and 75 µM phosphorus. Depicted are mean values ± SD (*n* = 3).

**Fig. 3:**
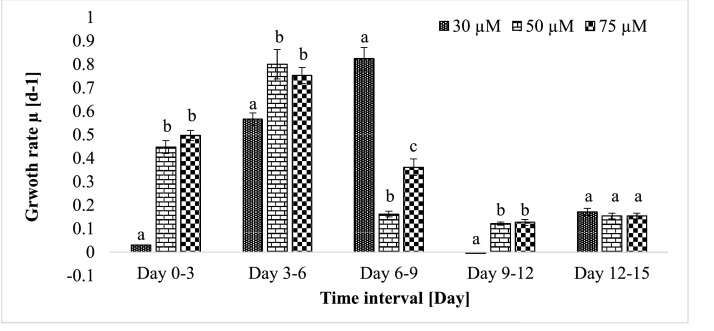
Growth rate of *M. aeruginosa* NIVA Cya 43 in 30, 50, and 75 µM phosphorus culture in different intervals of time. Shown are mean values ± SD (*n* = 3) determined at different time points. (Tukey's HSD after one-way ANOVA,<0.001). Values with different letters within each time span are significantly different (*p* < 0.05).

*M. aeruginosa* NIVA Cya 43 was cultivated under varying phosphorus concentrations, with the experiment featuring 75 µM phosphorus having an initial cell concentration of 8.33 × 10^4^ ± 0.008 cells/mL. Finally, the highest cell concentration was observed in treatment 75 µM which was 21.04 × 10^6^ ± 0.65 cells/mL where 17.73 × 10^6^ ± 1.40 cells/mL and 9.14 × 10^6^ ± 0.40 cells/mL cells concentrations were observed in treatment 50 µM and 30 µM, respectively. Growth rates exhibited fluctuations, with the most significant growth occurring between days 3 and 6, the value showed 0.752 d^−1^. In the 50 µM phosphorus experiment, the initial cell concentration was 9.17 × 10^4^ ± 0.030 cells/mL, and similar growth patterns were observed, with the most substantial growth observed between days 3 and 6, the value showed 0.8 d^−1^. In the 30 µM phosphorus experiment, characterized by lower phosphorus concentrations, the initial concentration was 9.16 × 10^4^ ± 0.016 cells/mL, and the highest growth rate was observed from day 6 to 9, the value showed 0.82 d^−1^ (Figs. 2 and 3).

### 3.2 Chymotrypsin inhibitors in *M. aeruginosa* NIVA Cya 43 in different phosphorus cultures

**Fig. 4:**
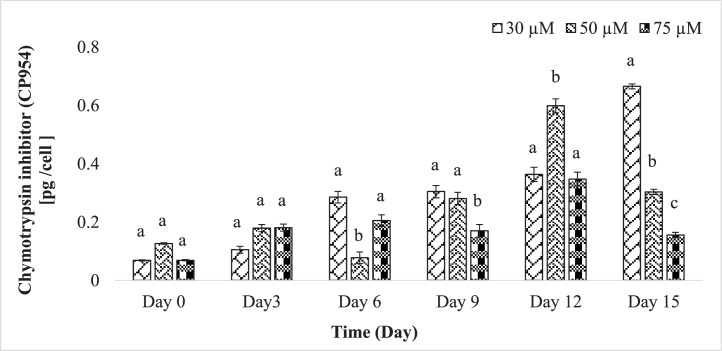
Content of the chymotrypsin inhibitor CP954 per cell in 30 µM, 50 µM and 75 µM phosphate experiment. Shown are mean values ± SD and *n* = 3.(Tukey's HSD after one-way ANOVA, D0 pCP954 = 0.333, D3 pCP954 =0.046, D6 pCP954 = 0.002, D9 pCP954 = 0.004, D12 pCP954 = 0.002, D15 pCP954 <0.001). Values with different letters within each time point are significantly different (*p* < 0.05).

**Fig. 5:**
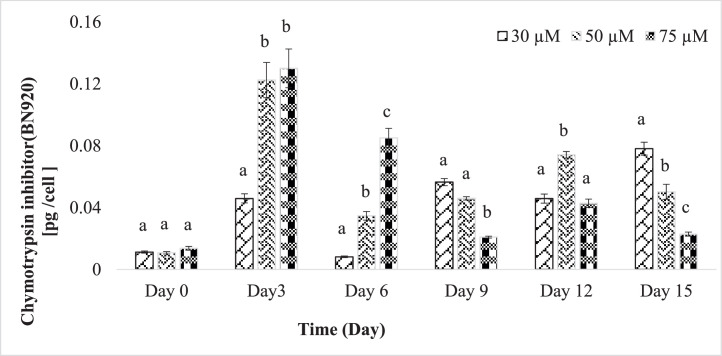
Content of the chymotrypsin inhibitor BN920 per cell in 30 µM,50 µM, and 75 µM phosphate experiment. Shown are mean values ± SD and *n* = 3. (Tukey's HSD after one-way ANOVA, D0 pCP954 = 0.792, D3 pCP954 <0.001, D6 pCP954 = 0.002, D9 pCP954 = 0.0190, D12 pCP954 = 0.002, D15 pCP954 = <0.001). Values with different letters within each time point are significantly different (*p* < 0.05).

Chymotrypsin inhibitors, specifically CP954, data displayed a wide range of concentrations, varying from 0.069 to 0.665 pg/cell, with the highest levels observed on day 15 (Fig. 4). In contrast, BN920 ranged from 0.014 to 0.13 pg/cell, peaking on day 3 (Fig. 5). Notably, lower phosphorus content correlated with higher CP954 concentrations, while BN920 exhibited the opposite trend.

**Fig. 6:**
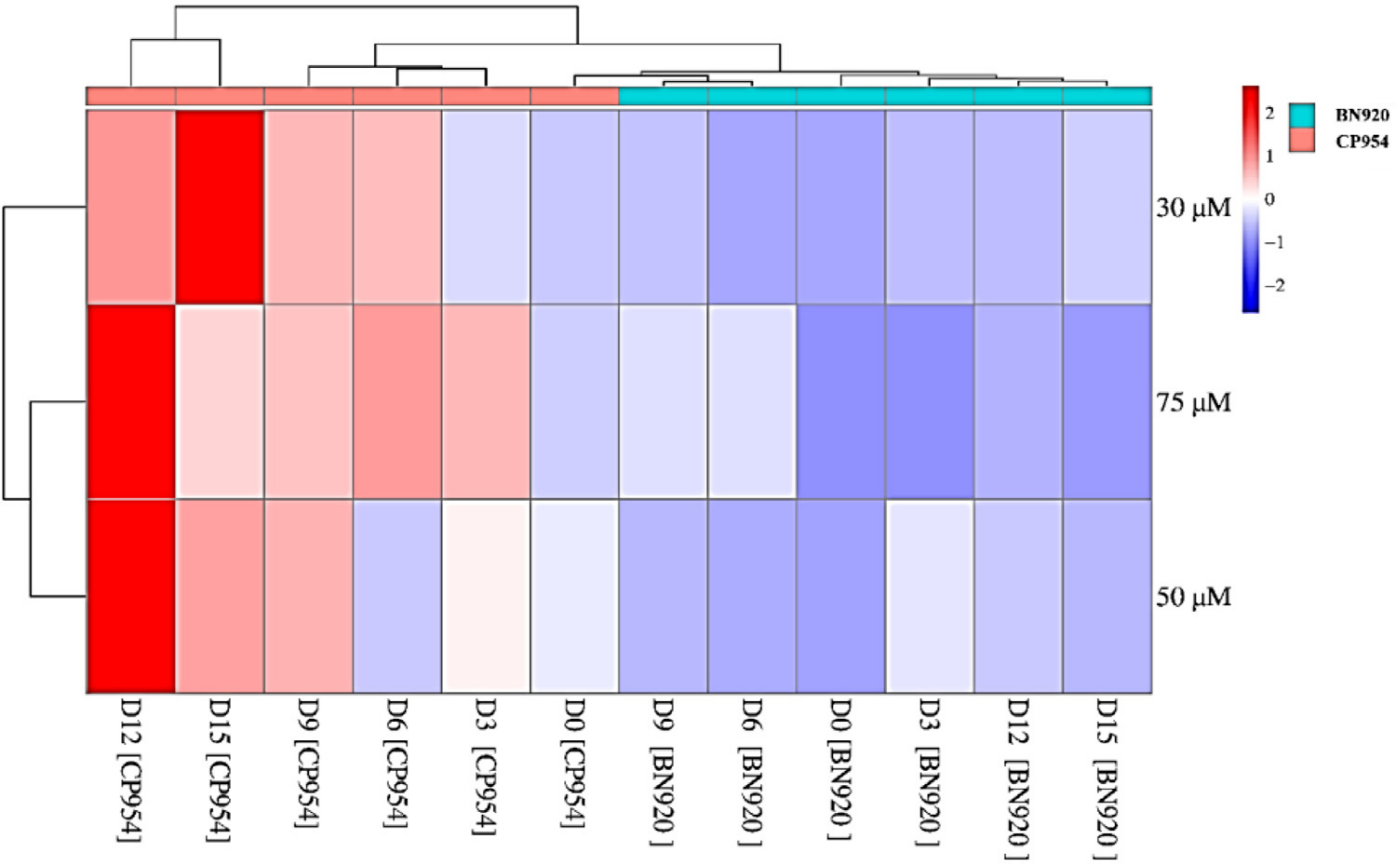
Visualizing chymotrypsin inhibitors (CP954 and BN920) with heatmap.

Comparing three distinct experiments, it was evident that the 30 µM phosphorus experimental data yielded the highest CP954 inhibitor content and growth rate, outperforming other treatments (Fig. 6). Interestingly, the impact of phosphorus on cell abundance diminished towards the experiment's end. When the initial phosphorus concentration was 50 µM or 75 µM, the highest growth rates were observed between days 3 and 6. In contrast, the 30 µM experimental data showed the peak growth rate occurring between days 6 and 9. Notably, the 30 µM experimental data exhibited the highest CP954 content on day 15, while the 50 µM and 75 µM experiments had lower concentrations of this inhibitor per cell. This suggests that decreasing phosphorus content leads to an increase in CP954 concentration, while the reverse trend is observed for BN920 content.

## 4 Experimental Design, Materials and Methods

### 4.1 Culturing conditions and experimental organisms

#### 4.1.1 Preparation of wright's cryptophytic (WC) medium

*M. aeruginosa* NIVA Cya 43 (NORCCA, Norwegian Institute for Water Research, Oslo, Norway) was cultured with WCmedium. [1], [2], [3], [4]. The standard WC medium with a phosphorus concentration of 50 µM was created by mixing 5 ml of stock solutions CaCl_2_×2H_2_O, MgSO_4_×7H_2_O, NaHCO_3_, NaNO_3_, Na_2_EDTA, FeCl_3_×6 H_2_O, CuSO_4_×5H_2_O, ZnSO_4_×7H2O, CoCl_2_×6H_2_O, and MnCl_2_×4H_2_O with 4.5 L of ultra-pure water, and then filling up to a total volume of 5 L with ultra-pure water. To prepare phosphorus-reduced medium (30 µM), specific volumes of stock solution K_2_HPO_4_×3H_2_O were added, along with 5 ml of the other previously prepared solutions. For the 30 µM phosphorus medium, 3 ml of K2HP04×3H2O was added, while for the 75 µM phosphorus medium, 7.5 ml of K2HP04×3H20 was used. To maintain osmotic conditions in the medium, 8.44 ml of KCl was added to the 30 µM phosphorus medium. The standard 50 µM phosphorus medium was prepared by mixing 5 ml of K2HP04×3H20 with 5 L of ultra-pure water.

#### 4.1.2 Growth of *M. aeruginosa* NIVA Cya43 in different phosphorus medium

*M. aeruginosa* was cultivated in batch culture (Biozentrum, Universität zu Köln, Germany), and subsequently, the optical density of the pre-cultured cyanobacterial strain was measured at 470 nm using a spectrophotometer (Macherey & Nagel, PF-11). Subsequently, the cyanobacterial culture was inoculated into 300 ml conical flasks, each containing 100 ml of autoclaved WC medium, all conducted under sterile conditions. These flasks were placed on a horizontal shaker (90 rpm) under continuous light conditions (46 µmol photons×m^−2^×s^−1^) at a room temperature of 20 °C. Cyanobacterial cell abundance was assessed daily through light microscopy. To achieve this, 0.5 ml subsamples were extracted from three randomly selected flasks under sterile conditions. The cyanobacterial cells were counted using a Neubauer-improved counting chamber (with a depth of 0.1 mm and an area of 0.0025 mm²). To ensure accuracy, a minimum of 100 cells per sample was counted. The experiment extended over 15 days, and population growth rates were calculated at 3-day intervals using a specific formula based on the cell abundance data.

$$\mu \;\left[d-1\right]=\frac{\ln\left(N_{t2}\right)-\ln\left(N_{t1}\right)}{\Delta t}$$

**Formula 1:** Population growth rate r of *M. aeruginosa* NIVA Cya 43. N_t1_ and N_t2_ represent the cell abundances [cells/ml] at the time points t1 and t2, and Δt is the time interval between t1 and t2 [d].

### 4.2 Extraction and quantification of chymotrypsin inhibitors by using LC-MS

Utilizing the methodology outlined by Hasan MM et al. [1], the meticulous extraction of chymotrypsin inhibitors from *M. aeruginosa* NIVA Cya 43 was carried out, followed by their quantification using an advanced setup that combined an Accela ultra-high-pressure liquid chromatography system (UHPLC, Thermo Fisher) with an Exactive Orbitrap mass spectrometer (Thermo Fisher). This integrated system, encompassing a 1250 psi pump, an autosampler, and a photodiode array detector (PDA) for comprehensive analysis, featured a C18-nucleosil column (EC 125/2 Nucleosil, 100–3; Macherey and Nagel, Düren, Germany) as the stationary phase in the chromatographic procedure. The chromatography employed a meticulously crafted gradient [1], consisting of ultra-pure water and acetonitrile (ACN), with both solvents containing 0.05% trifluoroacetic acid (TFA) to ensure the precision and reproducibility of results. In the mass spectrometry process, the instrument operated in the electrospray ionization mode (ESI), employing positive ionization and maintaining a constant temperature of 325 °C with a steady nitrogen gas flow. Furthermore, the capillary voltage was carefully set at 60 V, while the spray voltage was established at 4.5 kV. For optimal data acquisition, a scan range was defined within the range of 150 to 1500 Da, and all qualitative and quantitative analyses were conducted using the highly specialized Xcalibur software developed by Thermo Fisher.

During each run, a precisely measured 10 µL of the sample was meticulously injected, and each sample was subjected to duplicate measurements to ensure result reliability, constituting technical replicates. Under the specified conditions, BN920 eluted after 1.85 min, whereas CP954 eluted after 2.21 min [1].

To quantify the chymotrypsin inhibitors, present in the samples, a calibration curve-based approach was employed. These calibration curves were initially established using purified chymotrypsin inhibitors and the internal standard MC-LR. Specifically, the calibration curve for BN920 covered a range from 0 to 35 µg/ml, with a regression equation of *y* = 0.661777x - 0.207 and a high coefficient of determination (R² = 0.9908). Simultaneously, the calibration curve for CP954 spanned from 0 to 150 µg/ml, featuring a regression equation of *y* = 0.662427x + 3.66638, with a coefficient of determination (R² = 0.9621).

### 4.3 Statistical analysis

The statistical analyses were performed using Sigmaplot 11.0 and R Studio. The dataset was subjected to a one-way analysis of variance (ANOVA), followed by a post hoc analysis utilizing Tukey's honestly significant difference (HSD) test. Graphs were generated using Microsoft Office Excel 2007.

## Limitations

Microalgae and their properties are sensitive to its environment, physiological parameters and culture nutrient conditions [5]. Cyanobacterial growth is influenced by various environmental factors, including temperature and humidity, and the exact effects of these variables can vary depending on the specific species of cyanobacteria and environmental conditions; however, it is important to note that in this study, the effects of these environmental factors were not investigated.

## Ethical Statement

The experiment was conducted following guidelines, and the data were collected accordingly.

## Funding

We thank 10.13039/501100008001Universität zu Köln, Germany for this research.

## CRediT authorship contribution statement

**Md. Mohibul Hasan:** Conceptualization, Methodology, Software, Writing – review & editing. **Zahidul Islam:** Writing – review & editing.

## Data Availability

Data describing effects of different phosphorus concentrations on growth and chymotrypsin inhibitors in Microcystis aeruginosa NIVA Cya 43 using LC-MS (Original data) (Mendeley Data).
